# Mice haploinsufficient for *Map2k7*, a gene involved in neurodevelopment and risk for schizophrenia, show impaired attention, a vigilance decrement deficit and unstable cognitive processing in an attentional task: impact of minocycline

**DOI:** 10.1007/s00213-016-4463-y

**Published:** 2016-10-24

**Authors:** R.L. Openshaw, D.M. Thomson, J.M. Penninger, J.A. Pratt, B.J. Morris

**Affiliations:** 1 Institute of Neuroscience and Psychology, College of Medical, Veterinary and Life Sciences, University of Glasgow, West Medical Building, Glasgow, G12 8QQ UK; 2 Strathclyde Institute of Pharmacy and Biomedical Sciences, University of Strathclyde, Glasgow, G4 0RE UK; 3 Institute for Molecular Biotechnology of the Austrian Academy of Sciences (IMBA), 1030 Vienna, Austria

**Keywords:** Cognition, Attention, MKK7, JNK, MAP kinase

## Abstract

**Rationale:**

Members of the c-Jun N-terminal kinase (JNK) family of mitogen-activated protein (MAP) kinases, and the upstream kinase MKK7, have all been strongly linked with synaptic plasticity and with the development of the neocortex. However, the impact of disruption of this pathway on cognitive function is unclear.

**Objective:**

In the current study, we test the hypothesis that reduced MKK7 expression is sufficient to cause cognitive impairment.

**Methods:**

Attentional function in mice haploinsufficient for *Map2k7* (*Map2k7* ^*+/−*^ mice) was investigated using the five-choice serial reaction time task (5-CSRTT).

**Results:**

Once stable performance had been achieved, *Map2k7* ^*+/−*^ mice showed a distinctive attentional deficit, in the form of an increased number of missed responses, accompanied by a more pronounced decrement in performance over time and elevated intra-individual reaction time variability. When performance was reassessed after administration of minocycline—a tetracycline antibiotic currently showing promise for the improvement of attentional deficits in patients with schizophrenia—signs of improvement in attentional performance were detected.

**Conclusions:**

Overall, *Map2k7* haploinsufficiency causes a distinctive pattern of cognitive impairment strongly suggestive of an inability to sustain attention, in accordance with those seen in psychiatric patients carrying out similar tasks. This may be important for understanding the mechanisms of cognitive dysfunction in clinical populations and highlights the possibility of treating some of these deficits with minocycline.

**Electronic supplementary material:**

The online version of this article (doi:10.1007/s00213-016-4463-y) contains supplementary material, which is available to authorized users.

## Introduction

Considerable research efforts have focussed on the role of mitogen-activated protein (MAP) kinase signalling cascades in neuronal function. The importance of the MEK-ERK pathway for cognition has become particularly clear (Samuels et al. 2009), with the realisation that altered expression or activity of pathway components leads to neurodevelopmental disorders associated with intellectual disability (San Martin and Pagani 2014). In contrast, less attention has been directed at understanding the role of the c-Jun N-terminal kinase (JNK) pathway in neuronal function. In this pathway, three genes (*MAPK8–10*) encode isoforms of JNK (JNK1–3), which can be activated by either of the upstream kinases MKK4 or MKK7 (Wang et al. 2007; Coffey 2014). Mutations in JNK3 cause severe intellectual disability (Shoichet et al. 2006; Kunde et al. 2013), whilst sequence variations in the *MAP2K7* gene (encoding MKK7) are associated with prefrontal cortex dysfunction and cognitive impairment in schizophrenia (Winchester et al. 2012). This is consistent with experimental evidence that JNK1, MKK7 and MAP3K12 (a kinase upstream of MKK7) are all crucial for the development of the neocortex (Hirai et al. 2002; Hirai et al. 2011; Yamasaki et al. 2011; Riches and Reynolds 2014; Xu et al. 2014), that JNK1 (Li et al. 2007) and JNK2 (Chen et al. 2005) mediate aspects of synaptic plasticity in the mature mouse hippocampus and that MKK7 orthologue mutation impairs long-term memory in *Caenorhabditis elegans* (Lakhina et al. 2015). However, the impact of disruption of this pathway on specific cognitive domains is unclear.

Many psychiatric disorders, including schizophrenia (Green 2006), are partially characterised by a distinctive pattern of cognitive impairment that includes deficits in working memory and in the ability to sustain attention. Several neurochemical and metabolic changes observed in patients are centred on the prefrontal cortex, a region involved in multiple cognitive processes (Goldman-Rakic et al. 2000), including a crucial role in aspects of normal attentional function (Rossi et al. 2009). This raises the possibility that the decreased MKK7 expression, as observed in patients with schizophrenia (Winchester et al. 2012), contributes directly to cognitive impairment.

Attentional function can be investigated in mice by the five-choice serial reaction time task (5-CSRTT), a well-validated, translational, operant paradigm that was based on the continuous performance test (CPT) (Cornblatt et al. 1988; Nuechterlein et al. 2008; Robbins 2002; Humby et al. 1999). The CPT reveals attentional deficits in patients with neuropsychiatric disorders, for example, patients with schizophrenia (Suwa et al. 2004), bipolar disorder (Najt et al. 2005), including mania specifically (Sax et al. 1999), and ADHD (Epstein et al. 2003). The 5-CSRTT and CPT probe analogous behavioural and neural mechanisms in the rodent and human brain (Robbins 2002), which reinforces their relevance for the current NIMH Research Domain Criteria (RDoC) initiative to focus on fundamental domains of disease-relevant neural function (Casey et al. 2014).

Here, we test the hypothesis that reduced MKK7 expression is sufficient to cause cognitive impairment by investigating attentional function in mice haploinsufficient for *Map2k7* (*Map2k7* ^*+/−*^ mice) by using the 5-CSRTT. We also test the ability of minocycline to improve any deficits seen in the 5-CSRTT, as minocycline is currently showing promise in clinical trials as an adjuvant therapy for the treatment of cognitive and negative symptoms of schizophrenia (reviewed in Chaves et al. 2015). Specifically, available data suggest that minocycline treatment improves attentional performance in patients (Liu et al. 2014).

## Materials and methods

### Subjects

Mice heterozygous for a functional *Map2k7* gene (*Map2k7* ^*+/−*^) were produced by replacement of a portion of exon 9 with a PGK-Neo cassette as described in Sasaki et al. (2001) and have been backcrossed onto the C57Bl6 mouse strain. For the 5-CSRTT, 16 *Map2k7* ^*+/−*^ mice (7 female, 9 male) and 16 wild-type (WT) (7 female, 8 male) littermates were used, 18.9 ± 0.6 weeks of age at the start of the study, weighing 26.5 ± 0.7 g. All mice had experienced no previous procedures (naïve to drugs and testing) and were singularly housed in a temperature and humidity-controlled room (21 °C, 45–65 % humidity) with a 12-h light/dark cycle (lights on at 08:00). Mice were food restricted to 85–90 % of their free-feeding weight and had ad libitum access to water throughout the experiment. Testing was carried out daily between 09:00 and 17:00, Monday to Friday and in accordance with the Animals (Scientific Procedures) Act 1986.

### The five-choice serial reaction time task

In the 5-CSRTT, mice are required to respond via nose poke to brief flashes of light that appear pseudorandomly in five available holes for a large number of trials (100) in order to receive a liquid food reward from the magazine located in the wall opposite a curved wall containing the holes. If an incorrect response is made (poking a hole in which the stimulus did not appear) or a missed trial occurs (not responding within the limited hold (LH) period of 5 s), mice are punished by a timeout period (5 s) along with illumination of the main ‘house light’. Trials are separated by an inter-trial interval (ITI). Many aspects of their performance are automatically recorded (see below for more information) in order to detect differences in sustained, divided and spatial attention.

#### Apparatus

Eight mouse nine-hole operant chambers with dimensions of 12 by 13 cm (Campden Instruments Ltd., Cambridge Cognition Limited) were used for the experiment, which were enclosed in separate noise attenuation outer cabinets with a ventilator fan providing low-level, constant background noise. Mice were allocated an operant box randomly and were always tested in the same operant box throughout the experiment. Nine circular holes are evenly spaced along a curved side, of which four holes (holes 2, 4, 6 and 8) were blocked off leaving five available for use in the task. The operant chambers were controlled by Campden BNC Control software.

#### Performance measures

The performance measures for each session analysed were the following:

- % Accuracy—calculated by the formula (number of correct responses / number of correct responses + number of commission errors) × 100
- % Omissions—calculated by the formula number of missed trials / total number of trials completed
- Number of commission errors (nose poke during the LH period into a hole where the stimulus had not been presented)
- Number of premature responses (nose poke during the ITI period)
- Number of perseverative responses (repeat nose poke following a correct response, in either the correct hole or another hole, before collecting the food reward earned)
- Number of food magazine entries during the ITI
- Total number of trials completed
- Latency to make a correct response
- Mean reward collection latency
- Latency to consume reward (the duration the mouse was in the food magazine for the first magazine entry following reward delivery)
- Vigilance decrement (the extent to which performance declined over the course of each session. Calculated by subtracting the no. omissions data from final 20 trials completed from the first 20 trials completed by each mouse, for each session)
- Intra-individual variability of correct response times (IIV; the variability of response times for each mouse over the course of each session. Calculated by the standard deviation of response times for each mouse per session)
- Intra-individual variability of incorrect response times
- Overall variability of correct response times—calculated by the standard deviation of response times over the course of five sessions
- Overall variability of incorrect response times

#### Habituation and training to high and stable performance

The protocol was according to our standard procedures (Thomson et al. 2011). Mice were first habituated to the liquid reinforcer (Yazoo® Strawberry Milkshake) and operant boxes before undergoing 10 stages of training, which took 65 daily sessions in total.

As stage 1–10 progress, the length of time for which the stimulus is lit (stimulus duration 32–1 s) and LH (the length of time the mice have to respond within, from the beginning of the presentation of the stimulus 37–5 s) decrease and the criteria become more stringent (>30 correct − >50 correct + >80 % accuracy) until the mice are performing at a level deemed appropriate to see differences in other aspects of performance measures (e.g. % omissions) without being confounded by differences in task ability (Bari et al. 2008).

Once each individual mouse had reached criteria of the final training stage 10, they were rested without daily training, whilst mice not at criteria of stage 9 continued. Mice on rest were given a reminder training session twice per week and if they fell below criteria on a reminder session, they were trained daily until criteria were reattained. This training regime is encouraged for operant-based training (Oomen et al. 2013) because ‘over’training the mice that learn the task more quickly than others could increase variability in performance and have confounding effects on the interpretation of the results.

Once all mice reached the final training stage, they moved onto full test conditions which were the same as stage 10 apart from the ITI which was not fixed at 5 s; instead, it pseudorandomly varied between 2, 5, 10 or 15 s (vITI). This prevents the use of a temporal strategy to complete the 5-CSRTT (see Bari et al. 2008). As the overall ITI length was increased, the maximum session duration was increased from 30 to 45 min. They were trained on the vITI until their performance stabilised (which took 12 sessions), and the final 5-day stable data were taken as ‘baseline’ performance.

#### Minocycline administration

Mice received minocycline in their normal drinking water (0.5 mg/ml; protected from light; Sigma-Aldrich Co. M9511, St. Louis, MA, USA) for a 7-day period. Fresh minocycline solution was prepared every second day and provided at room temperature. The dose and duration of the minocycline administration were chosen to reflect, as closely as possible, the treatment protocols that are associated with symptomatic improvement in patients with schizophrenia. Administration of minocycline to mice in drinking water at 0.5 mg/ml, as used here, produces a brain concentration of around 2 μM (Smith et al. 2003), which is equivalent to the CSF concentrations achieved in humans during standard antibacterial dosing regimes (Macdonald et al. 1973; Agwuh and McGowan 2006). This administration regime in mice is similar to that of McKim et al. (2016). In the psychiatric context, improvement in patient symptoms has been noted with 3 days of minocycline treatment at equivalent doses (Miyaoka 2012). Mice were therefore tested on the 5-CSRTT on days 4 and 7 after the start of minocycline treatment. Consumption of minocycline-treated water was monitored daily for each mouse; they each received an average of 81.6 ± 3.1 mg/kg/day of minocycline.

### Western blotting

Total protein from prefrontal cortex and hippocampal tissue was extracted from male and female mice as follows: Approximately 20 mg tissue was homogenised in RIPA buffer (10 mM Tris-HCL pH 7.4, 150 mM NaCl, 1 mM EDTA pH 8, 0.5 % *w*/*v* NP40, 0.1 % *w*/*v* SDS, 0.1 % *w*/*v* sodium deoxycholate), protease inhibitor cocktail (Sigma, P8340) and 1 M phosphatase inhibitor (Na_3_VO_4_). Cellular extracts were then centrifuged at 10,000 RPM for 10 min at 4 °C and supernatant collected. Protein concentrations of each individual sample were determined by using a Bradford protein assay and normalised. Protein was denatured by heating to 80 °C for 10 min in sample buffer (NuPAGE, Novex, NP0007) and sample reducing agent (NuPAGE, Novex, NP0004) before being subjected to SDS-PAGE in 10 % Bis-Tris gel (NuPAGE, Novex, NP0302BOX) and transferred to Invitrolon PVDF membrane (Novex, LC2005). Membranes were placed in TTBS buffer (120 mM Tris-HCL pH 7.5, 150 mM NaCl, 0.05 % Tween-20) supplemented with 3 % skimmed milk and blocked for 30 min at room temperature. Membranes were then incubated with total MKK7 (tMKK7) primary antibody (1:10,000, Genetex, #103563) overnight at 4 °C with constant agitation. The next day, they were washed 3 × 10 min in TTBS and then incubated with anti-rabbit secondary antibody (1:10,000) for 90 min at room temperature with constant agitation. Blots were then washed once in TTBS, then 2 × 10 min in TBS. Membrane-bound secondary antibodies were detected by using Chemiluminescent HRP Substrate (Immobilon, Millipore, WBKLS0100), and digital images of Western blots were captured by PXi4 (Syngene). Blots were then reprobed with Actin-HRP antibody (1:12,000, Santa Cruz, sc-1616) overnight at 4 °C with constant agitation and washed 3 × 10 min in TTBS. Actin specific bands were detected by using ECL reagent (Signalfire, Cell Signalling Technologies, 68835). Digital images of blots were quantified by using ImageJ software, with tMKK7 values normalised to Actin values.

### Statistical analysis

All statistical analyses were carried out by using Minitab 16 Statistical Software. In all cases, results were considered significant if *p* < 0.05. All data are presented as mean ± standard error of the mean (SEM). Graphs were created by using GraphPad Prism 3.03.

#### 5-CSRTT data

One significantly atypical wild-type mouse was removed from the study after initial training stage 10 because of consistent abnormal repetitive behaviour (hyperactive rotational movements) that prevented the mouse from completing the task properly, disguising its true cognitive ability.

Comparison of the last 5-day stable performance between two experimental groups is a method generally utilised to examine group differences (Sanchez-Roige et al. 2012): The last 5 days of well-trained, stable performance were analysed between genotypes and, where appropriate, were compared with performance on days 4 and 7 of the 7-day minocycline treatment.

Unless stated otherwise, results were analysed by using a two-way repeated measures ANOVA with daily session as a within subject factor, genotype as a between subject factor and each individual mouse nested within genotype. Minocycline treatment data were analysed by a three-way repeated measures ANOVA with session (i.e. day of minocycline treatment) and treatment as within subject factors, genotype as a between subject factor and each individual mouse nested within genotype. Before data from males and females were grouped, effects of gender were investigated and were non-significant for all measurements excluding correct response latency. Therefore, ‘gender’ was included in the statistical analysis as a between subject factor for this measure. Post hoc tests were conducted by using Tukey’s method for multiple comparisons where appropriate. Anderson-Darling tests for normality were carried out on all data, and if required, data were Box-Cox transformed to the optimal λ; however, where means are reported, untransformed data are used.

#### Western blot data

ImageJ data were analysed by a one-tailed or two-tailed *t* test between genotypes.

## Results

### *Map2k7*^+/−^ mice exhibit impaired attention in the 5-CSRTT

**Fig. 1:**
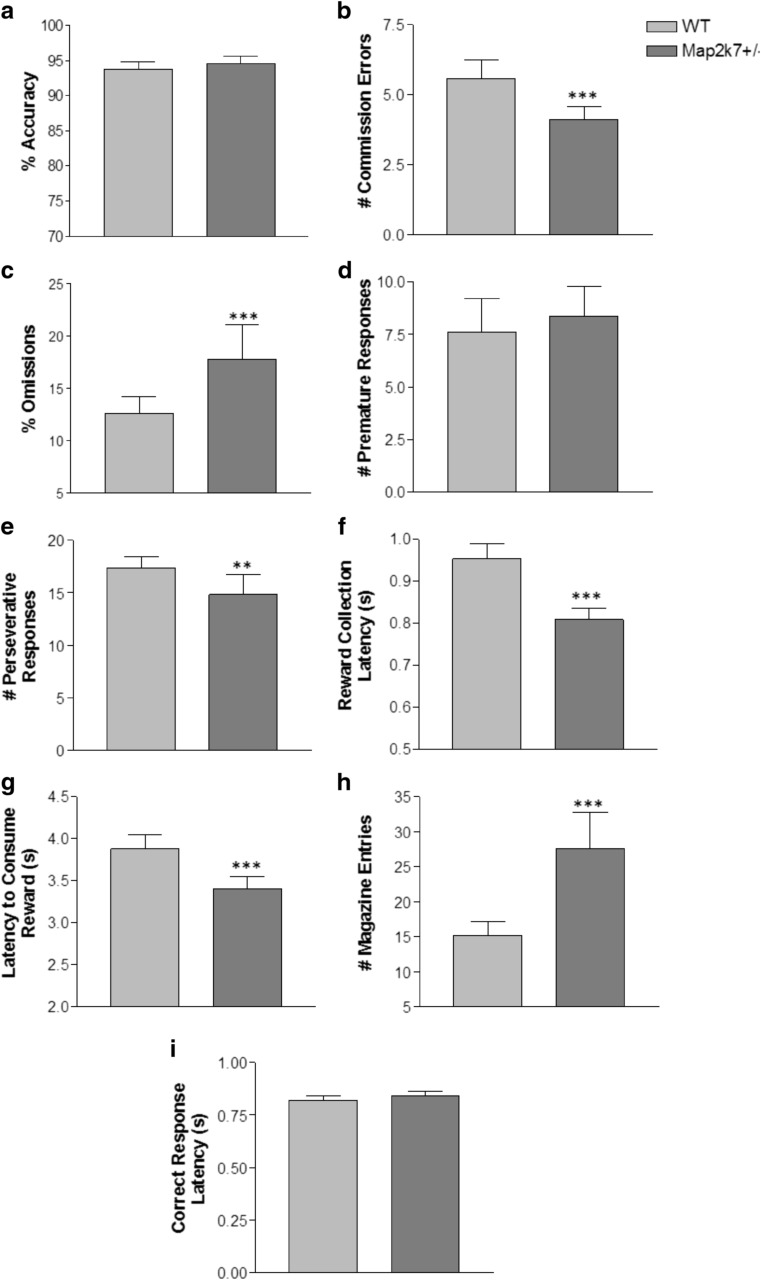
*Map2k7* ^*+/−*^ mice show an attentional deficit in the 5-CSRTT which is not due to impairments in motivation and/or motor ability. *Map2k7* ^*+/−*^ mice perform with similar accuracy (**a**) and make slightly fewer commission errors (**b**) than WTs, miss more trials (**c**) and display no inhibitory control deficit (**d**, **e**). *Map2k7* ^*+/−*^ mice display decreased reward collection (**f**) and consumption (**g**) latencies, make more entries into the reward magazine (**h**) and have similar correct response latencies (**i**) compared to WTs. Each *bar* represents the average of the last 5-day stable performance in the test stage. Data analysed by a two-way repeated measures ANOVA (with daily session as a within subject factor, genotype as a between subject factor and each individual mouse nested within genotype) with Tukey’s post hoc and are presented as mean ± standard error of the mean (SEM). **p < 0.01; ***p < 0.001, *N*_WT_ = 15, *N*_*Map2k7+/−*_ = 16

**Fig. 2:**
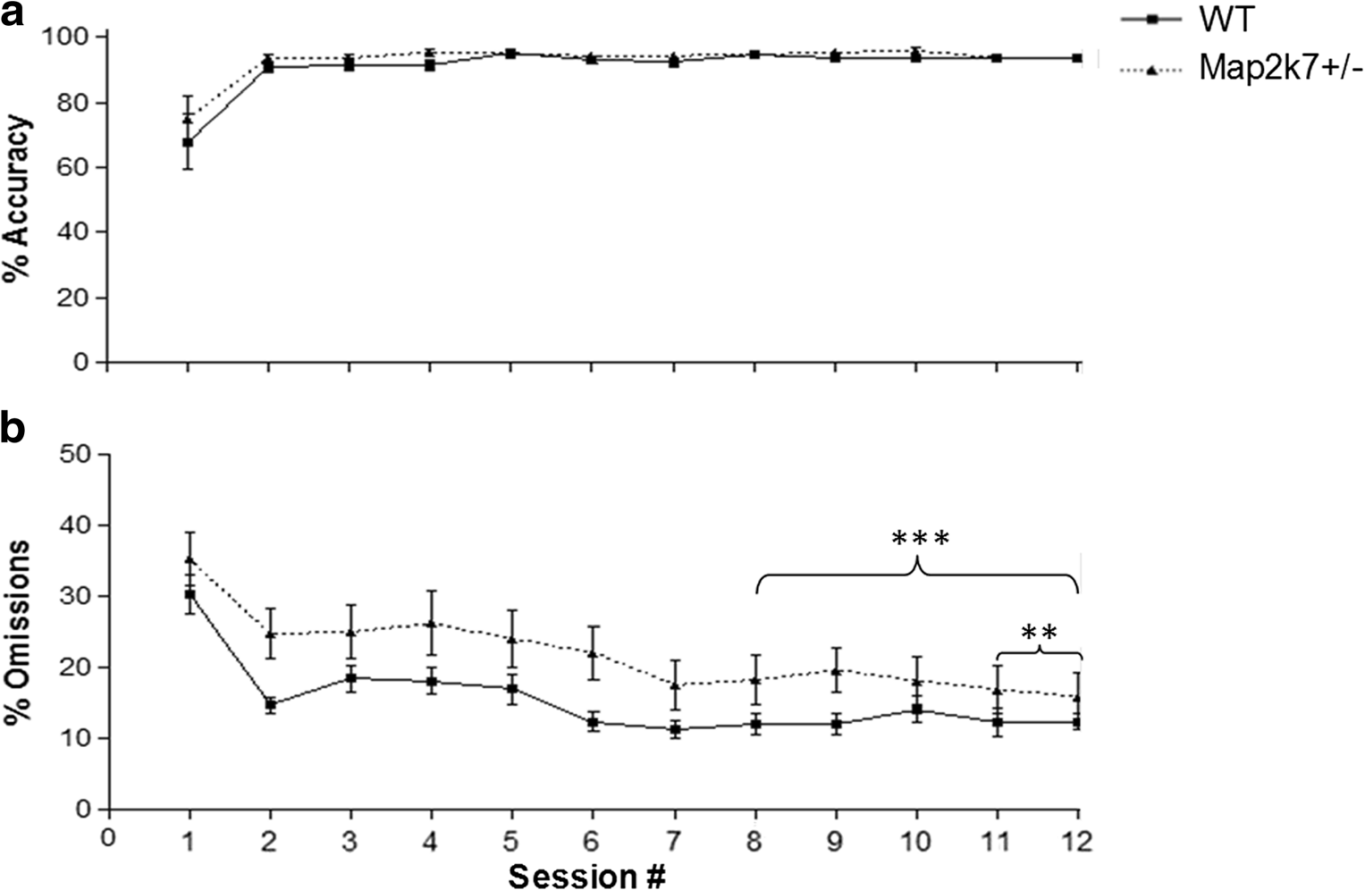
*Map2k7* ^*+/−*^ mice consistently show a similar level of accuracy (**a**) and a higher percentage of missed trials (**b**) than WT mice over each vITI training session of baseline performance (the last 5-day vITI training). The significant effect of genotype on % omissions is also observed when analysing the last 2 days of baseline performance rather than the last 5 (*F*_(1, 29)_ = 8.71; *p* = 0.006). *Numbers along the х-axis* represent session numbers from the first session of vITI training following basic training stages 1–10. Data analysed by a two-way repeated measures ANOVA (with daily session no. as a within subject factor, genotype as a between subject factor and each individual mouse nested within genotype) with Tukey’s post hoc and are presented as mean ± standard error of the mean (SEM). **p < 0.01, ***p < 0.001 (genotype effect), *N*_WT_ = 15, *N*_*Map2k7+/−*_ = 16

Throughout training and once stable performance had been attained, *Map2k7* ^*+/−*^ mice consistently showed a similar level of accuracy as WT mice (Figs. 1a and 2a); in fact, they performed marginally better at 94.6 ± 0.9 % accuracy compared to WT littermates at 93.5 ± 0.7 % (*F*_(1, 116)_ = 3.87; *p* = 0.052) and made fewer commission errors overall (*Map2k7* ^*+/−*^ 4.063 ± 0.67; WT 5.33 ± 0.48) (*F*_(1, 116)_ = 17.24; *p* = 0.0001) (Fig. 1b). Even when challenged with shorter stimulus durations, *Map2k7* ^*+/−*^ mice were still able to perform to a similar extent to WT mice (Supplementary Fig. S1). However, *Map2k7* ^*+/−*^ mice showed impaired attentional performance, as indicated by an elevated number of omissions made compared to wild-type littermates (*F*_(1, 116)_ = 42.36; *p* = 0.0001) (Fig. 1c). Moreover, this was consistent throughout training on the vITI (Fig. 2b). The significant effect of genotype on % omissions was also observed when analysing the last 2 days of baseline performance rather than the last 5. Inhibitory control measures showed that *Map2k7* ^*+/−*^ mice were not impaired compared to WTs: They exhibited a similar number of premature responses (Fig. 1d) and made significantly fewer perseverative responses (*F*_(1, 116)_ = 12.13; *p* = 0.001) (Fig. 1e). However, *Map2k7* ^*+/−*^ mice were quicker to collect (*F*_(1, 116)_ = 240.97; *p* = 0.0001) and consume (*F*_(1, 116)_ = 228.13; *p* = 0.0001) the reward (Fig. 1f, g), completed almost all trials (99.6 ± 0.4 trials completed on average over the 5-day baseline, compared to WTs completing all 100) and had similar correct response latencies to WTs (*F*_(1, 135)_ = 1.51; *p* = 0.22) (Fig. 1i), showing good motivation to perform the task. *Map2k7* ^*+/−*^ mice also entered the reward magazine more frequently during the ITI period (i.e. when there is no reward there to collect) than WTs (*F*_(1, 116)_ = 51.92; *p* = 0.0001) (Fig. 1h). There were also no significant differences between WTs and *Map2k7* ^*+/−*^ mice for trials or sessions to criteria for initial acquisition (just prior to vITI training) of the 5-CSRTT (Supplementary Fig. S2). Collectively, these results indicate that *Map2k7* ^*+/−*^ mice exhibit an attentional deficit displayed as increased omissions in the 5-CSRTT. Furthermore, this deficit is not due to incapability of learning or carrying out of the task, as shown by good accuracy and initial task acquisition. The deficit is also not due to motivational/motoric impairment: they displayed clear motivation to perform the task in several aspects of performance, and they had slightly faster reward collection and consumption latencies, which indicate that measurements such as the number of omissions are not confounded for reasons such as being physically incapable of reaching the stimulus during the limited hold period.

### *Map2k7*^+/−^ omission deficit shows signs of being improved by minocycline

**Fig. 3:**
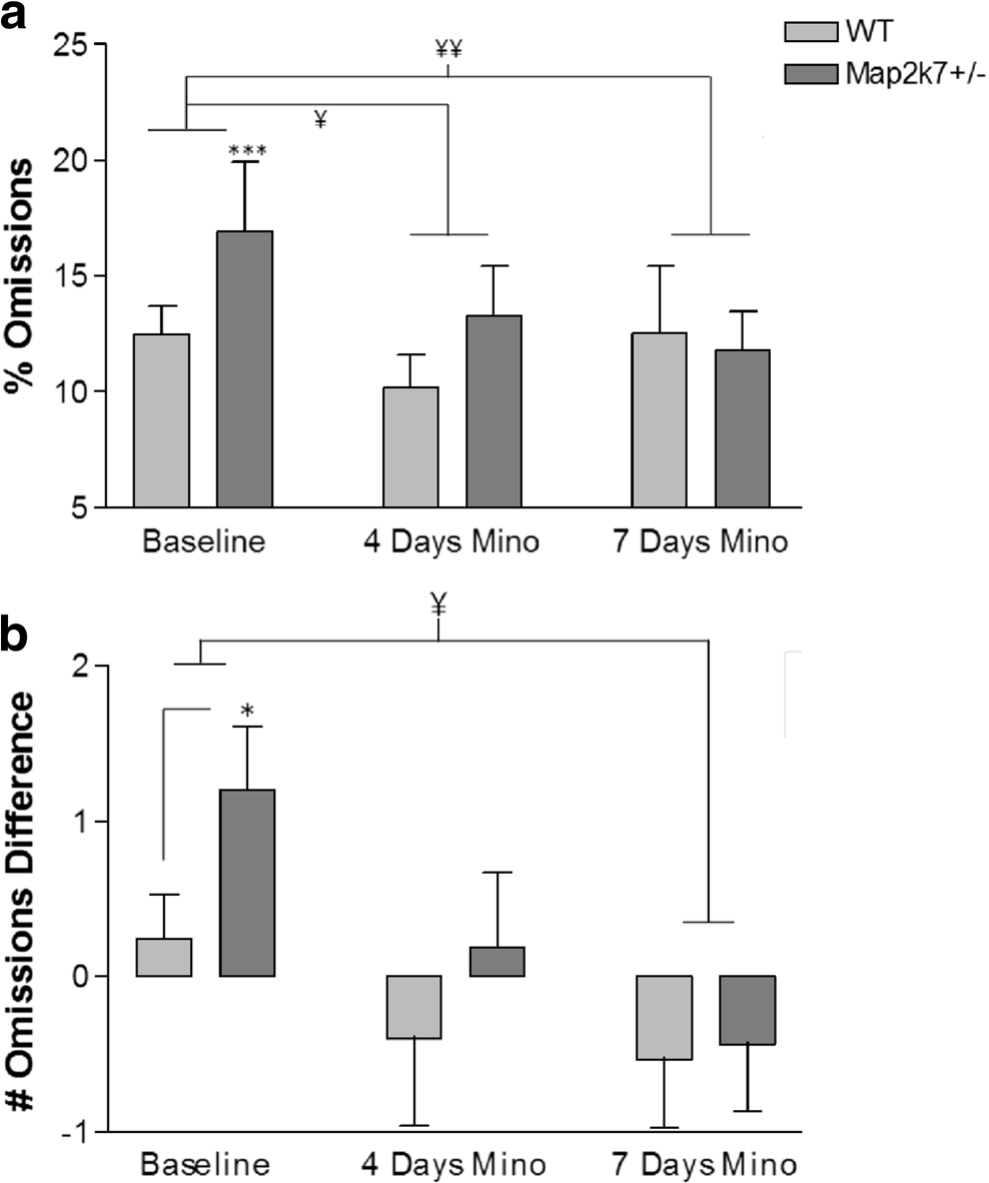
**a** Minocycline shows signs of improving the % omissions deficit seen by *Map2k7* ^*+/−*^ mice. When the data from the 2 days of minocycline are analysed along with the 5-day baseline, there is a significant effect of session (*F*_(6, 182)_ = 2.57; *p* = 0.02) and the significant effect of genotype is no longer there (*F*_(1, 182)_ = 0.224; *p* = 0.224). Mice received 81.6 ± 3.1 mg/kg/day minocycline on average. Data analysed by a three-way repeated measures ANOVA with daily session and minocycline treatment as within subject factors, genotype as a between subject factor and each individual mouse nested within genotype with Tukey’s post hoc and are presented as mean ± standard error of the mean (SEM). ***p < 0.001 (vs. baseline WT); ¥*p* < 0.05, ¥¥p < 0.01 (minocycline vs. baseline), *N*_WT_ = 15, *N*_*Map2k7+/−*_ = 16. **b** *Map2k7* ^*+/−*^ mice display a sustained attentional deficit to a larger extent than WTs, reflected throughout the last 5-day stable performance (baseline) by an increased proportion of number of omissions occurring during the last 20 % of the trials as compared to the first 20 % of each daily session. Minocycline showed signs of improving the vigilance decrement in mice by the seventh day of treatment. The difference score was calculated by subtracting data collected during the first 20 trials completed from data collected during the final 20 trials completed by each mouse, for each daily session. Data were then analysed by either a two-way repeated measures ANOVA with daily session as a within subject factor, genotype as a between subject factor, with each individual mouse nested within genotype (data from Baseline), or as a one-way ANOVA between genotypes (data from days 4 and 7 of treatment). Data are presented as mean ± standard error of the mean (SEM). *p < 0.05 (vs. baseline WT); ¥*p* < 0.05 (minocycline vs. baseline), *N*_WT_ = 15, *N*_*Map2k7+/−*_ = 16

Minocycline is a tetracycline antibiotic which is currently showing promise in clinical trials for treatment of the negative and cognitive symptoms of schizophrenia (reviewed in Chaves et al. 2015). Overall, minocycline, administered for 7 days, improved the % omissions score for both *Map2k7* ^*+/−*^ and WT mice by the seventh day of treatment (effect of session *F*_(3, 80)_ = 4.53; *p* = 0.006), by which time the significant genotype difference at baseline had disappeared (Fig. 3a). Furthermore, mice committed fewer omissions in the 5-CSRTT by the seventh day of minocycline treatment than they had ever achieved before the treatment (from 16.9 ± 1.7 at baseline compared to 11.8 ± 2.9 by the seventh day of minocycline treatment). Indeed, when performance over the five baseline and two minocycline sessions was assessed, a significant effect of session was detected (*F*_(6, 182)_ = 2.57; *p* = 0.02), without an overall significant effect of genotype (*F*_(1, 182)_ = 0.224; *p* = 0.224), and the two minocycline sessions were both significantly different from each of the five baseline sessions (Fisher’s post hoc test). Minocycline also had the effect of removing the statistical significance of other phenotype-specific changes in performance, such as the number of commission errors and perseverative responses, the latency to consume reward and the number of magazine entries, but not for reward collection latency (Supplementary Fig. S3).

### Map2k7^+/−^ mice display a deficit in ability to sustain attention

For each daily session, the number of omissions performed by each mouse in the first 20 trials they completed was subtracted from those performed during the final 20 trials to give a difference score that shows the extent to which performance for each mouse declines with session progress, which is known as a ‘vigilance decrement’ (Parasuraman et al. 1987; Robbins 2002). On average, *Map2k7* ^*+/−*^ mice show a vigilance decrement compared to WT mice (who did not) at baseline, manifesting as a significantly higher increase in the number of omissions at the end of each session than the beginning, compared to WT mice (*F*_(1, 144)_ = 4.56; *p* = 0.034) (Fig. 3b). Minocycline improved the number of omission difference score by the seventh day of treatment overall, whilst, again, removing the statistical significance between genotype groups (Fig. 3b; effect of session: *F*_(2, 182)_ = 4.66; *p* = 0.02).

### Map2k7^+/−^ mice are more varied in their response times than WT mice

**Fig. 4:**
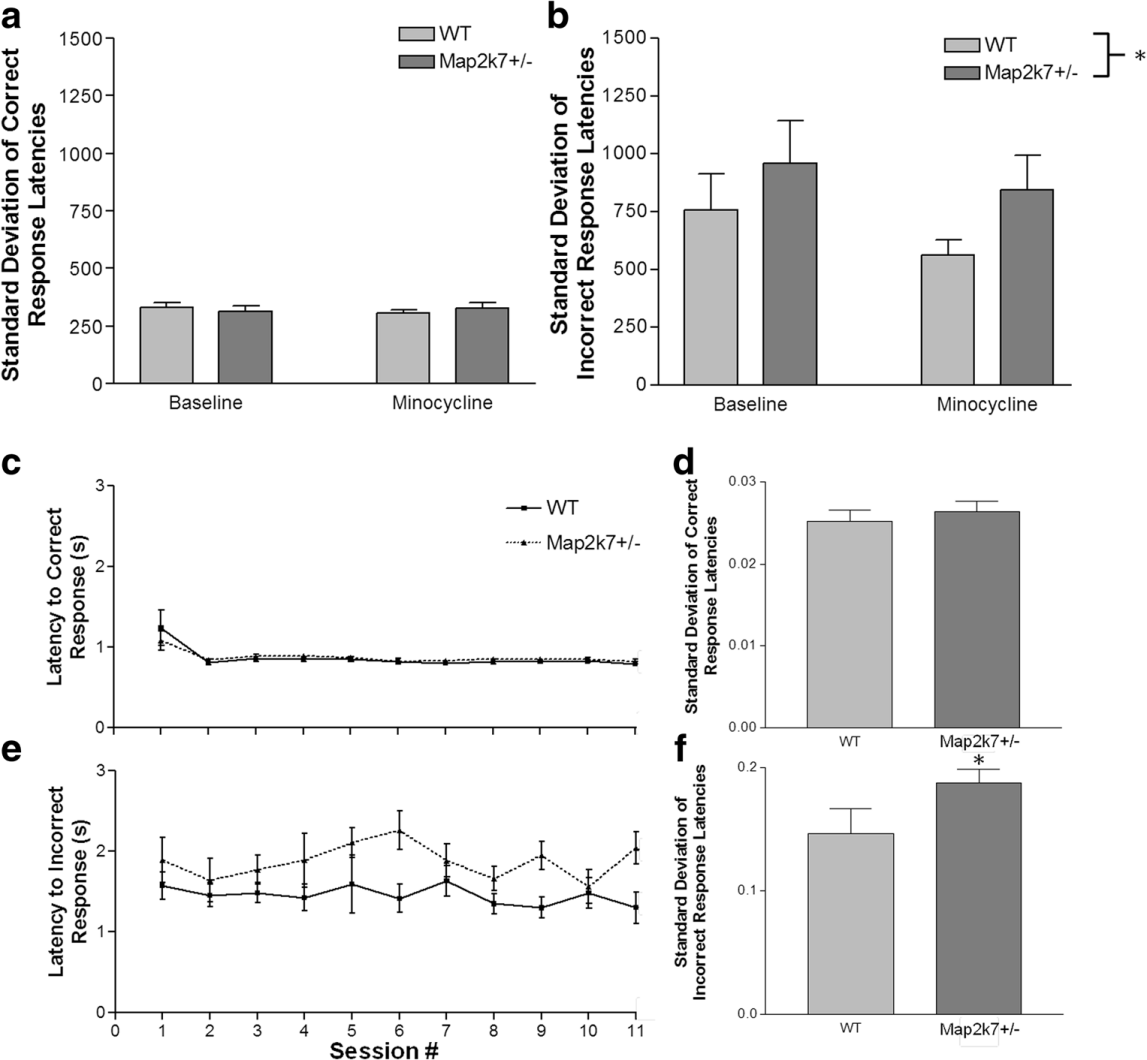
*Map2k7* ^*+/−*^ mice have a higher intra-individual reaction time variability for incorrect, but not correct, response times than WT mice throughout baseline that was not significantly alleviated by minocycline treatment (**a**, **b**). Increased variability also occurred for *Map2k7* ^*+/−*^ mice compared to WTs on average on a day-to-day basis, seen during training to a vITI and baseline performance for latency to incorrect (**e**) but not correct responses (**c**). **d**, **f** The standard deviations of each group over the course of the last 5-day stable performance (baseline). Data was analysed by either a two-way repeated measures ANOVA with daily session as a within subject factor, genotype as a between subject factor, with each individual mouse nested within genotype (data from **a** to **e**), or a one-tailed *t* test between genotypes (data from **d** and**f**). Data are presented as mean ± standard error of the mean (SEM). *p < 0.05 (vs. WT), *N*_WT_ = 15, *N*_*Map2k7+/−*_ = 16

Intra-individual reaction time variability (IIV) is a measure of variability in response times of a subject carrying out a task over the course of a single session, thus quantifying short-term fluctuations in an individual’s performance, and gives an indication of the stability of cognitive processing (Kanai and Rees 2011). IIV is perturbed in a number of neuropsychiatric disorders including schizophrenia (Geurts et al. 2008; Kaiser et al. 2008). We assessed IIV by measuring the variability of reaction times of each mouse to make a correct and incorrect response over the course of each daily session for every day of baseline (stable) performance. Interestingly, *Map2k7* ^*+/−*^ mice show significantly higher variability in their reaction times when making incorrect responses (Fig. 4b; *F*_(1, 125)_ = 4.58; *p* = 0.034) but not when making correct responses (Fig. 4a; *F*_(1, 144)_ = 0.48; *p* = 0.491). This effect was maintained when looking at group reaction time variability over the course of the vITI training, including the 5-day stable performance (Fig. 4c, e): Incorrect response times of *Map2k7* ^*+/−*^ mice vary significantly more on a day-to-day basis than WTs (Fig. 4e, f; *F*_(1, 4)_ = 6.43; *p* = 0.049), and group reaction time variability for correct responses remained the same for all mice (Fig. 4c, d; *F*_(1, 4)_ = 1.13; *p* = 0.453). Minocycline did not have a significant effect on the standard deviations of either correct or incorrect response times of mice compared to baseline performance (Fig. 4a, b).

### Map2k7^+/−^ mice have decreased tMKK7 in the prefrontal cortex and hippocampus

**Fig. 5:**
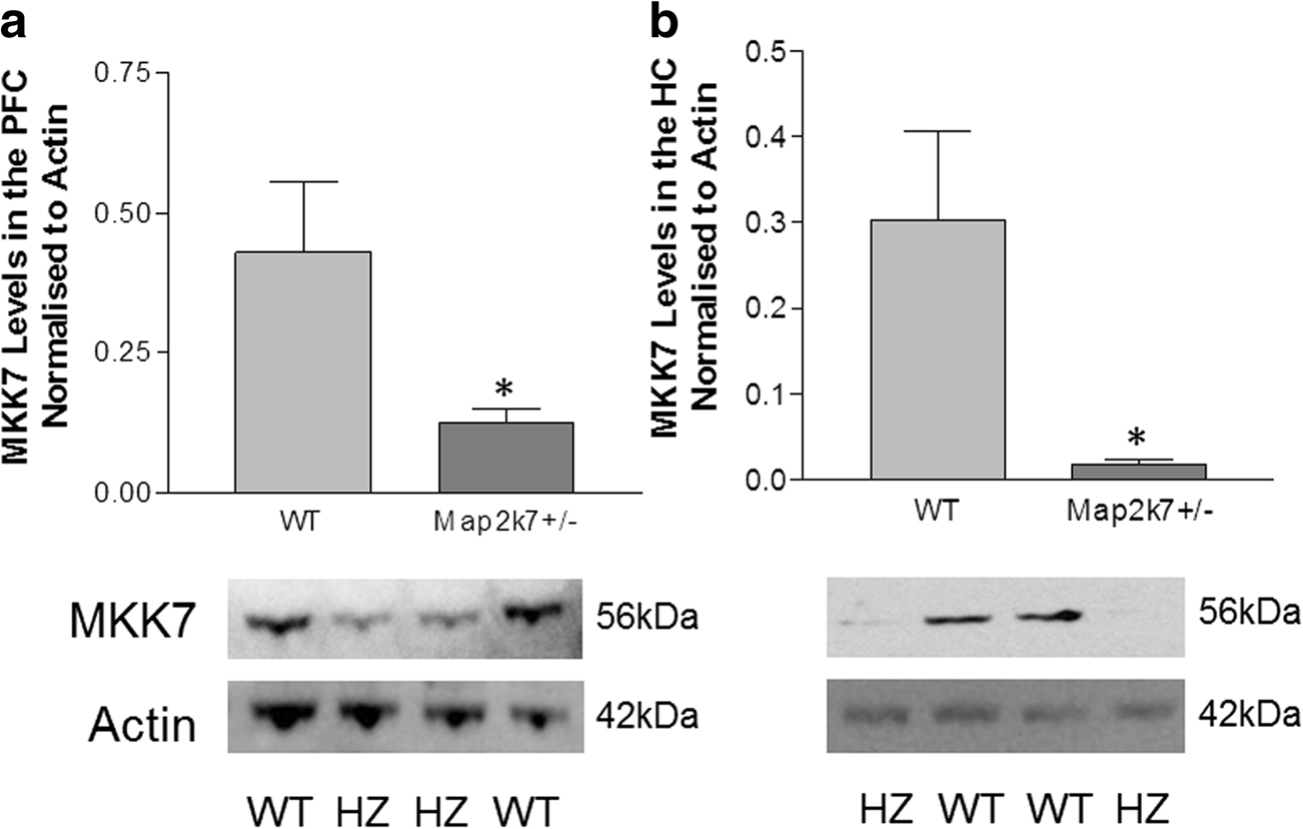
Western blot data show mice heterozygous (HZ) for the *Map2k7* gene have significantly decreased levels of MKK7 protein in the prefrontal cortex (**a**) and hippocampus (**b**). *WT* wild type, *HZ* Map2k7^+/−^ mice. Data analysed for PFC and HC separately by one-way ANOVA. Data are presented as mean ± standard error of the mean (SEM). PFC *p < 0.05 (vs. WT), *N*_WT_ = 6, *N*_*Map2k7+/−*_ = 6. HC *p < 0.05 (vs. WT), *N*_WT_ = 10, *N*_*Map2k7+/−*_ = 9

Having established behavioural differences in *Map2k7* ^*+/−*^ mice compared to their wild-type littermates, we investigated whether their heterozygosity is reflected in tMKK7 protein levels in the brain. We were particularly interested in the prefrontal cortex and hippocampus because of their well-established involvement in the cognitive deficits of schizophrenia (Tregellas et al. 2014; Harrison 1999). The prefrontal cortex (PFC) is required for attentional control in mice in general (Kim et al. 2016) and for normal performance in the 5-CSRTT (Robbins 2002); hippocampal activity is correlated with rat performance in the 5-CSRTT (Barbelivien et al. 2001), and hippocampal-prefrontal interactions have an essential role in cognition and behaviour in psychiatric disease (reviewed in Sigurdsson and Duvarchi 2016). Whole protein was extracted from prefrontal cortex and hippocampal tissue, and Western blotting confirmed a significant reduction of 56 kDa MKK7 (ɣ isoform) in both brain regions (Fig. 5; prefrontal cortex *F*_(1, 10)_ = 6.32, *p* = 0.031; hippocampus *F*_(1, 17)_ = 5.52, *p* = 0.031).

## Discussion

Mice haploinsufficient for the *Map2k7* gene display an attentional deficit in the 5-CSRTT that shows signs of being alleviated by minocycline, as well as an increased vigilance decrement and intra-individual reaction time variability. These deficits may reflect the decreased MKK7 protein levels in the prefrontal cortex and hippocampus in *Map2k7* ^*+/−*^ mice. Overall, *Map2k7* haploinsufficiency causes behavioural alterations in accordance with those seen in psychiatric patients carrying out similar tasks, along with molecular changes relevant to cognition and psychiatric disorders.

### Map2k7^+/−^ mice have decreased MAP2K7 protein in the prefrontal cortex and hippocampus

Western blotting shows 56 kDa MKK7 (gamma isoform) to be significantly decreased in the prefrontal cortex and hippocampus of *Map2k7* ^*+/−*^ mice compared to their wild-type littermates. Decreased MKK7 in the PFC and hippocampus (HC) is likely to have a disruptive effect on behaviour in the 5-CSRTT because hippocampal-prefrontal interactions occur in various cognitive and behavioural functions, and disruption of the PFC and HC is consistently implicated in psychiatric disease (reviewed in Sigurdsson and Duvarci 2016). It is interesting that a highly conserved SNP genetically associated with schizophrenia is located immediately upstream of an alternatively spliced exon only present in the MAP2K7ɣ isoform (Winchester et al. 2012). In the current study, although it is possible that MAP2K7 may be decreased throughout the whole brain, the reductions of MAP2K7ɣ in the PFC and HC are likely to have functional implications which may explain the deficits seen in the 5-CSRTT.

### Map2k7^+/−^ mice display an attentional deficit

Once stable performance had been attained, accuracy in the 5-CSRTT was unimpaired in *Map2k7* ^*+/−*^mice, indicating that they are able to acquire the basic principles of the task to normal levels of performance. Equally, there was no evidence that these mice showed increased levels of impulsivity (premature responses) or compulsivity (perseveration), and response latencies were slightly faster, indicating good motivation to perform the task. Strikingly, there was a very specific deficit in that rates of missed responses were substantially raised. The combination of results (increased omissions, good accuracy, faster response latencies) suggests that *Map2k7* ^*+/−*^ mice are unable to maintain the same levels of attention as WT littermates, despite the fact that they appear to be highly motivated and understand how to carry out the task (Robbins 2002; Humby et al. 1999). Changes in accuracy levels as opposed to omissions are frequently looked at as the main measure of attentional function in the 5-CSRTT; however, several studies confirm that increased omissions with the absence of an accuracy deficit probably result from stimulus detection failures as a consequence of inattention, so long as motoric/motivational impairments can be ruled out (Cordova et al. 2006; Fletcher et al. 2007; Inglis et al. 2001; Risbrough et al. 2002; Young et al. 2004, 2007; Tzanoulinou et al. 2015).

As well as omission deficits, *Map2k7* ^*+/−*^ mice also display a slight vigilance decrement that manifests as an increased amount of missed trials at the end of each session than at the beginning, compared to WT mice, whose performance did not decline over the course of a session. Patients with schizophrenia consistently show a similar vigilance deficit (Nestor et al. 1990, 1991; Hahn et al. 2012; Mass et al. 2000; Lysaker et al. 2010; Young et al. 2013), as do other rodent models relevant to schizophrenia (Barnes et al. 2012, 2014).

*Map2k7* ^*+/−*^ mice also showed another form of attention deficit: increased intra-individual reaction time variability (IIV). IIV is a measure of variability in response times of a subject carrying out a task over the course of a single session. Originally seen merely as ‘noise’ in experimental data, researchers now realise that it also reflects the stability of cognitive processing and short-term fluctuations in performance over a session (Kaiser et al. 2008) and it has been suggested that IIV, as well as average task performance levels, are good predictors for real-world functioning (Stuss et al. 2003). IIV is consistently increased in schizophrenia (Kaiser et al. 2008), ADHD (reviewed in Kuntsi and Klein 2012) and several other psychiatric/cognitive disorders (Musso et al. 2015; Camicioli et al. 2008; Geurts et al. 2008). Moreover, it is under investigation as a reliable predictor for those who are at risk of developing schizophrenia (Shin et al. 2013), ADHD (Henrίquez-Henrίquez et al. 2015) and even for cognitive function in itself (Grand et al. 2016). Here, we looked at the distribution of response times for each mouse over the course of each daily session to give a measure of IIV. *Map2k7* ^*+/−*^ mice have increased IIV for incorrect responses but not correct responses. They also respond more variably on a day-to-day basis as a group when making incorrect responses compared to WT mice. IIV is an intriguing indicator of cognitive function because of its sensitivity, reliability and robustness across different tasks that involve reaction times (Kuntsi and Klein 2011). Establishing the underlying neural mechanisms to increased IIV have been the focus of many studies which have shown that increased IIV is correlated with disruption of dopamine regulation in the PFC and subsequent increase of neural signal to noise (MacDonald et al. 2006, 2009; Stefanis et al. 2005). Nevertheless, other neural systems, hitherto unexplored, are likely to be involved.

In addition to *Map2k7* ^*+/−*^ mice showing increased variability in making incorrect responses, they also show altered latencies in other measurements. They are quicker or similar in all other latency measurements recorded: *Map2k7* ^*+/−*^ mice are faster to collect and consume the reward and respond just as quickly as WT mice when making a correct response. As well as showing high motivation to complete the task, this set of results suggests that when the *Map2k7* ^*+/−*^ mice have noticed the stimulus, they are just as quick to respond correctly, but when they miss a stimulus (probably due to inattention), they have slower processing times than WTs before deciding to take a guess. This may manifest as increased, and more variable, incorrect response reaction times.

*Map2k7* ^*+/−*^ mice also make an increased number of magazine entries during the ITI period compared to WTs. The ITI is the period of time after they have collected the reward (if they responded correctly) or after the timeout period (if they responded incorrectly or missed the stimulus). Therefore, *Map2k7* ^*+/−*^ mice are showing signs of anticipating wrongly when they should receive a reward. Two possible explanations for this are either because they are applying an increased amount of salience to the reward or that they ‘like’ it more than WT mice. Previous studies in our lab have suggested that *Map2k7* ^*+/−*^ mice do not experience increased preference for sucrose (Thompson 2013); *Map2k7* ^*+/−*^ mice may therefore apply more salience to the reward magazine than WTs. Furthermore, *Map2k7* ^*+/−*^ mice appear to exhibit greater entrainment to the light stimulus as they do not show decreased accuracy in conjunction with their increased % omissions (Amitai and Markou 2010). Throughout any given trial, if they fail to detect the stimulus, instead of guessing which hole to poke (thus decreasing their % accuracy score), they withhold responding and consequently present with a missed trial. The decreased numbers of commission errors made by *Map2k7* ^*+/−*^ mice are also indicative of this. Frequent and mistaken trips to the reward magazine and strong entrainment to the light stimuli may both be examples of *Map2k7* ^*+/−*^ mice applying increased salience to some aspects of the task, which is interesting in relation to psychiatric disorders that include cognitive impairment, in particular schizophrenia, because one of the symptoms in patients is applying too much salience to particular, often irrelevant, aspects of the environment (Kapur 2003).

Overall, *Map2k7* ^*+/−*^ mice display a specific attentional deficit which is unlikely to be explained by lack of motivation or locomotor impairment. Although the molecular mechanisms involved in decreased hippocampal MKK7 of *Map2k7* ^*+/−*^ mice and how this results in impaired attention are currently unclear, future molecular research will glean more information about this.

### Minocycline shows signs of improving some aspects of performance in the 5-CSRTT

Minocycline is a semi-synthetic tetracycline antibiotic showing promise in current clinical trials for the treatment of the negative and cognitive symptoms of schizophrenia; importantly, it has shown ability to improve attentional deficits (Liu et al. 2014). It is an ideal candidate for schizophrenia treatment because it is already deemed safe for human consumption and readily crosses the blood-brain barrier (Zink et al. 2005). Minocycline was administered to *Map2k7* ^*+/−*^ mice for 1 week in their drinking water, with testing on the 5-CSRTT on days 4 and 7 of the treatment. Minocycline improved the % omissions score of the mice overall, but the *Map2k7* ^*+/−*^ mice in particular showed signs of continual improvement of their % omissions score throughout minocycline treatment, performing better on the seventh day of the treatment than they had ever performed beforehand. Although minocycline treatment did not have a significant effect on the IIV of *Map2k7* ^*+/−*^ mice, in the same way as it affected % omissions, the treatment improved performance of all mice overall with respect to the vigilance decrement, including having a particular influence on *Map2k7* ^*+/−*^ mice. Minocycline also had this effect on other phenotype-specific changes in performance, such as the number of commission errors and perseverative responses, latency to consume reward and the number of magazine entries. As minocycline has previously been shown to improve attention deficits in human patients with schizophrenia (Liu et al. 2014), these results further warrant the refinement of *Map2k7* ^*+/−*^ mice as a model of attentional and possibly other cognitive impairments because the ability to maintain and focus attention enhances performance in other cognitive domains.

Minocycline has also been shown to improve reaction times in healthy volunteers in a sustained attentional task (Sofuoglu et al. 2011). Additionally, in a mouse model relevant to schizophrenia produced by administration of an *N*-methyl-D-aspartate receptor (NMDAR) antagonist, MK801, minocycline improved deficits in prepulse inhibition and visuospatial memory (Levkovitz et al. 2007) and also improved phencyclidine-induced cognitive deficits in mice (Fujita et al. 2008). Despite minocycline having been shown to have beneficial effects in both healthy and pathological (cognitive deficits) human and rodent studies, the exact mechanism of action of minocycline is still unknown, although two main mechanisms have been proposed with relation to cognition: inhibition of activated microglia and/or enhancing glutamate release via NMDARs (Liu et al. 2014; Lisiecka et al. 2015). It is entirely conceivable that either or a combination of both of these mechanisms are relevant in the current study because of the potential for them both to interact with the MKK7/JNK pathway. The JNK pathway is essential for pro-inflammatory functions of microglia (Waetzig et al. 2005), and NMDARs are located upstream of the MKK7-JNK pathway (Centeno et al. 2006), suggesting that altering microglia and/or NMDAR activation states via minocycline has potential to affect regulation of the MKK7/JNK pathway in order to produce a cognitive enhancing effect. More molecular and clinical evidence on minocycline’s potential as an agent to improve cognition is needed. However, our data support the concept that this drug is effective in improving some aspects of attentional function.

### Conclusion

The results presented here demonstrate the importance of MKK7-JNK signalling for attentional processes. *MAP2K7* sequence variants show a strong genetic association with schizophrenia (Winchester et al. 2012), and other kinases closely involved in MKK7-JNK signalling have been detected as potentially associated with schizophrenia in recent GWAS (Morris and Pratt 2014; Schizophrenia Working Group of the Psychiatric Genomics 2014). In summary, mice haploinsufficient for the *Map2k7* gene show deficits in attention, a core cognitive impairment in many neuropsychiatric diseases (Millan et al. 2012), and show signs of improvement in attentional performance with minocycline treatment. Importantly, dissection of attentional processes revealed impaired vigilance and impaired cognitive stability, particularly when attending to incorrect responses which are highly relevant to ‘real-world’ functioning. Hence, the data may be important for understanding the mechanisms of cognitive dysfunction and highlight the possibility of treating some of these deficits with minocycline.

## Electronic supplementary material

ESM 1 (PDF 143 kb)
