# Supplementary material for: Mice haploinsufficient for Map2k7, a gene involved in neurodevelopment and risk for schizophrenia, show impaired attention, a vigilance decrement deficit and unstable cognitive processing in an attentional task: impact of minocycline
Source: Psychopharmacology (Berl). 2016 Oct 24;234(2):293–305. doi: 10.1007/s00213-016-4463-y (PMC5203862; doi:10.1007/s00213-016-4463-y)
Supplement: Supplementary file 1 — (PDF 143 kb) [file 213_2016_4463_MOESM1_ESM.pdf]

Supplementary Material

Mice haploinsufficient for *Map2k7*, a gene involved in neurodevelopment and risk for schizophrenia, show impaired attention, a vigilance decrement deficit and unstable cognitive processing in an attentional task: impact of minocycline

R.L. Openshaw[1], D.M. Thomson[2], J.M. Penninger[3], J.A. Pratt[2], B.J. Morris[1]

[1]University of Glasgow, Institute of Neuroscience and Psychology, College of Medical, Veterinary and Life Sciences, West Medical Building, G12 8QQ, Glasgow, United Kingdom

[2]University of Strathclyde, Strathclyde Institute of Pharmacy and Biomedical Sciences, G4 0RE, Glasgow, United Kingdom

[3]IMBA, Institute for Molecular Biotechnology of the Austrian Academy of Sciences, 1030 Vienna, Austria

Corresponding authors: **R.L. Openshaw and B.J. Morris**

Email: Brian.Morris@glasgow.ac.uk

University of Glasgow, Institute of Neuroscience and Psychology, College of Medical, veterinary and Life Sciences, West Medical Building, G12 8QQ, Glasgow, UK.

This work was supported by the Medical Research Council.

Additional Data

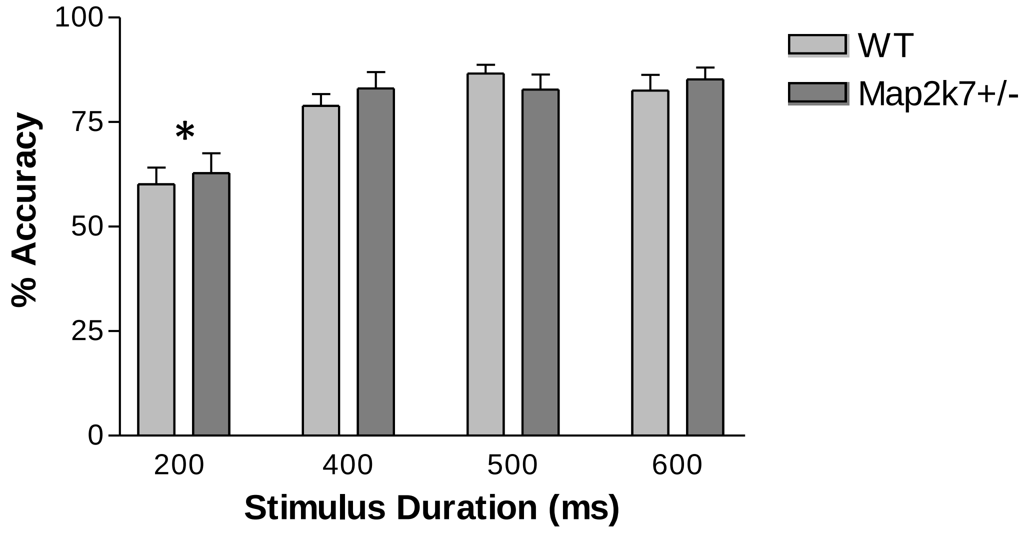

Supplementary Figure S1. Performance of *Map2k7*$^{+/-}$ mice in the 5-CSRTT remained similar to WTs prior to being challenged with shorter stimulus durations (SD). Throughout a single session, mice were subjected to variable stimulus durations with a fixed ITI of 5 seconds. Stimulus durations varied pseudorandomly across the session between 0.2, 0.4, 0.5 and 0.6 seconds in order to challenge accuracy. Data analysed by a repeated measures ANOVA, with genotype as a between subjects factor, stimulus duration as a within subjects factor and each individual mouse nested within genotype, with Tukey's post-hoc. Data are presented as mean ± SEM. Accuracy significantly decreased for all mice with stimuli of 0.2 seconds, but was maintained at a high level for 0.4, 0.5 and 0.6 second stimuli (effect of stimulus duration: $F_{(3,84)}$=30.63, *p<0.001 (vs. 0.4, 0.5, 0.6s SD); no effect of genotype: p=0.47; N_{WT}=15, $N_{Map2k7+/-}$=16).

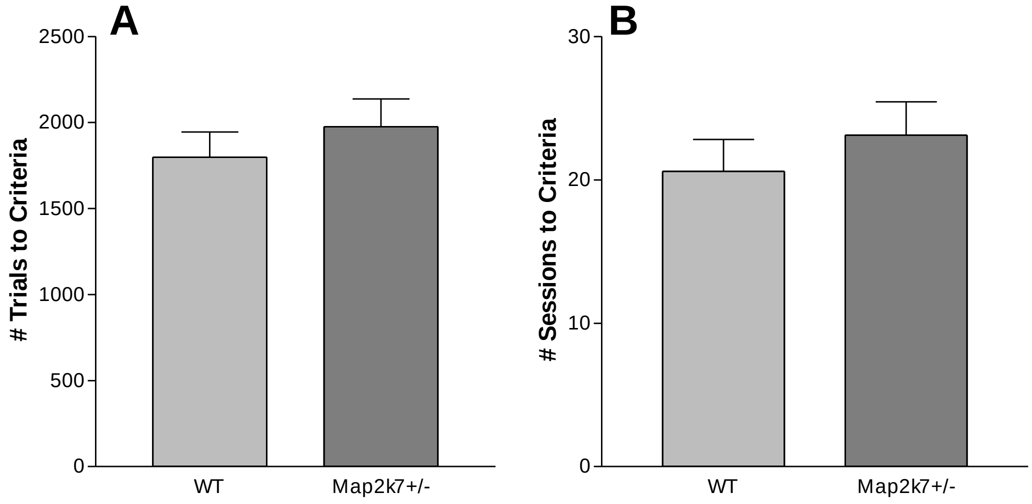

Supplementary Figure S2. WT and *Map2k7*$^{+/-}$ mice took a similar number of trials (**A**) and sessions (**B**) to reach criteria for initial acquisition of the 5-CSRTT. Initial acquisition occurred prior to introduction of the vITI, so the ITI was fixed at 5 seconds and stimulus duration fixed at 1 second. Data was analysed separately by a one-way ANOVA between genotypes. Data are presented as mean ± SEM. No significant effect of genotype: p=0.43 and p=0.39 for **A** and **B**, respectively. N_{WT}=15, $N_{Map2k7+/-}$=16.

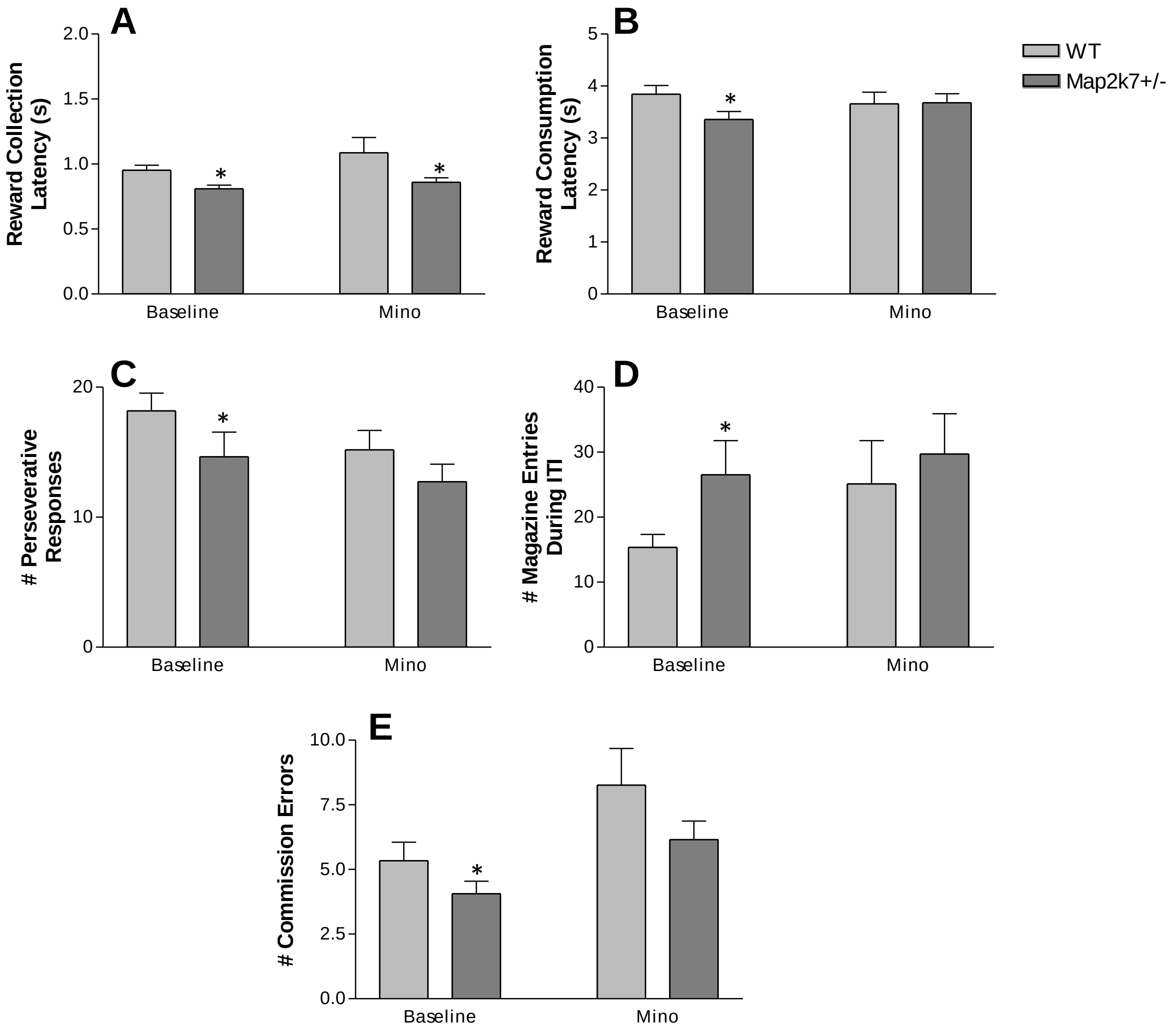

Supplementary Figure S3. Additional data on the effect of minocycline on other phenotype-specific changes in performance. Minocycline removed genotype statistical significance for: the latency to consume reward, the number of perseverative responses, magazine entries during the ITI and commission errors, but did not affect reward collection latency. Data analysed by a repeated measures ANOVA, with genotype as a between subjects factor, session as a within subjects factor and each individual mouse nested within genotype, with Tukey's post-hoc. Data are presented as mean ± SEM. *$p<0.05$ (vs. WT). N_{WT}=15, $N_{Map2k7+/-}$=16.
